# Supplementary material for: Diagnostic Yield of Genetic Testing for Ocular and Oculocutaneous Albinism in a Diverse United States Pediatric Population
Source: Genes (Basel). 2023 Jan 3;14(1):135. doi: 10.3390/genes14010135 (PMC9859104; doi:10.3390/genes14010135)
Supplement: Supplementary file 1 [file genes-14-00135-s001.zip › genes-2105397-supplementary.pdf]

Supplemental Table S1. Patient demographics and genetic testing results.

Patient #	Race and Ethnicity	Cutanous Findings	Ocular Findings	Family History	Genetic Test	Date of Test	Gene (Variant Classification)	Diagnostic Yield Positive
**1**	Other/ Hispanic	Yes	Nystagmus, fundus hypopigmentation, foveal hypoplasia	No	Albinism Panel (Dermatology) Plus	2021	*OCA2* (P)	No
**2**	White/NH	Yes	Nystagmus, fundus hypopigmentation, foveal hypoplasia	Yes (related to patient 3)	Albinism Panel (Dermatology) Plus	2019	*HPS5* (LP)	Yes
**3**	Other/NH	Yes	Nystagmus, foveal hypoplasia	Yes (related to patient 2)	Familial Variant Testing	2019	*HPS5* (LP)	Yes
**4**	Other/ Hispanic	Yes	Nystagmus, foveal hypoplasia, iris TID	No	DNA Analysis for Mutations in the Human HPS-1 and HPS-3 genes	2015	*HPS1* (LP/P)	Yes
**5**	White/NH	No	Nystagmus, foveal hypoplasia	No	Albinism Panel (Dermatology) Plus	2018	*TYR* (P), *TYR* (P), *LYST* (VUS)	Yes
**6**	Unknown	No	Nystagmus, fundus hypopigmentation, foveal hypoplasia, iris TID	No	Hypopigmentation Panel	2021	*HPS6* (LP)	Yes
**7**	Other/ Hispanic	Yes	Nystagmus, fundus hypopigmentation, foveal hypoplasia, iris TID	No	Oculocutaneous Albinism Panel	2019	*TYR* (P), *TYR* (P)	Yes
**8**	Black/NH	Yes	Nystagmus, fundus hypopigmentation, foveal hypoplasia, iris TID	Yes	Oculocutaneous Albinism Panel	2021	*SLC45A2* (P), *SLC45A2* (P)	Yes
**9**	Declined	Yes	Nystagmus, fundus hypopigmentation, foveal hypoplasia, iris TID	No	Whole Exome Sequencing	2020	*TYR* (P), *TYR* (P)	Yes
**10**	Other/ Hispanic	No	Nystagmus, foveal hypoplasia	Yes	GPR143 DNA Analysis	2009	No mutation	No
**11**	Other/ Hispanic	Yes	Nysatgmus, fundus hypopigmentation, foveal hypoplasia, iris TID	Yes	Albinism Panel (Dermatology) Plus	2021	*OCA2* (P), *OCA2* (LP)	Yes
**12**	Black/NH	Yes	Nystagmus, fundus hypopigmentation, foveal hypoplasia, iris TID	No	Albinism Panel (Dermatology) Plus	2020	*OCA2* (P), *OCA2* (LP)	Yes
**13**	Black/NH	Yes	None	No	Albinism Panel by Massively Parallel Sequencing	2015	*OCA2* (P)	No
**14**	White/NH	Yes	Nystagmus, fundus hypopigmentation, foveal hypoplasia, iris TID	No	Albinism Panel by Massively Parallel Sequencing	2020	*TYR* (P), *TYR* (P)	Yes
**15**	Black/NH	Yes	Nystagmus, fundus hypopigmentation, foveal hypoplasia, iris TID	Yes	OCA2 Targeted Deletion Analysis	2012	*OCA2* (LP/P), *OCA2* (LP/P)	Yes
**16**	Other/ Hispanic	No	Nystagmus, foveal hypoplasia, iris TID	No	Common Mutations in HPS1,HPS3/Hermansky-Pudlak Syndrome, TYR, OCA2	2006	*OA1/GPR143* (VUS->P (reclassification)), *TYR* (VUS/risk factor ->VUS/risk factor (reclassification))	No->Yes
**17**	Other/His panic	Yes	Nystagmus, fundus hypopigmentation, foveal hypoplasia	Yes	Hypopigmentation Panel	2021	*OCA2* (P), *OCA2* (VUS), *AP3D1* (VUS), *TYR* (VUS), *HPS5* (VUS)	Yes
**18**	Other/NH	Yes	Nystagmus, fundus hypopigmentation, foveal hypoplasia	No	Exomenext Select	2019	*OCA2* (P), *OCA2* (LP), *MC1R* (VUS)	Yes

**19**	White/NH	Yes	Nystagmus, fundus hypopigmentation, foveal hypoplasia	No	Invitae Inheirted Retinal Disorders Panel	2021	*OCA2* (P), *CNGA3* (VUS), *CYP4V2* (VUS), *GPR179* (VUS), *RP1* (VUS)	No
**20**	White/NH	Yes	Nystagmus, foveal hypoplasia	Yes	Oculocutaneous Albinism Panel	2020	*OCA2* (P), *OCA2* (LP), *TYR* (LP)	Yes
**21**	Native Hawaiian or Pacific Islander/NH	Yes	Nystagmus, fundus hypopigmentation, foveal hypoplasia, iris TID	No	Albinism Panel by Massively Parallel Sequencing	2014	*OCA2* (LP/P), *OCA2* (LP/P), *HPS5* (LP/P)	Yes
**22**	White/NH	Yes	Nystagmus, fundus hypopigmentation, foveal hypoplasia	No	Albinism Panel (Ophthalmology) Plus	2018	*TYR* (P), *TYR* (VUS)	Yes
**23**	Other/Hispanic	Yes	Nystagmus, foveal hypoplasia	No	NGS Pigmentation SmartPanel Casey Eye	2015	*TYRP1* (LP/P), *TYRP1* (LP/P)	Yes
**24**	White/NH	Yes	Nystagmus, fundus hypopigmentation, foveal hypoplasia, iris TID	Yes	Hermansky Pudlack and Oculocutaneous Albinism Panel	2015	*OCA2* (LP/P), *OCA2* (LP/P), *TYRP1* (LP/P), *LYST1* (nonsense mutation)	Yes
**25**	White/NH	Yes	Nystagmus, foveal hypoplasia	Yes	Albinism Panel (Dermatology) Plus	2018	*OCA2* (P), *OCA2* (LP)	Yes
**26**	White/NH	Yes	Nystagmus, fundus hypopigmentation	No	Albinism Panel (Dermatology) Plus	2018	*TYR* (VUS->VUS (reclassification))	No
**27**	Black/NH	Yes	Nystagmus	Unknown	Hermansky Pudlok Syndrome, Type I, Sequence	2008	No mutation	No
**28**	White/NH	Yes	Nystagmus, fundus hypopigmentation, foveal hypoplasia, iris TID	No	Oculocutaneous Albinism Panel	2021	*TYR* (P), *TYR* (VUS->P (reclassification)), *TYR* (VUS), *RAB27A* (VUS), *MC1R* (VUS)	No->Yes
**29**	Asian/NH	Yes	Nystagmus, fundus hypopigmentation, foveal hypoplasia	Unknown	Oculocutaneous Albinism Panel, 5 Genes, Casey Eye Institute	2012	*OCA2* (VUS->VUS (reclassification)), *OCA2* (VUS->VUS (reclassification))	No
**30**	White/NH	Yes	Fundus hypopigmentation, iris TID	No	Oculocutaneous Albinism Panel	2012	*TYR* (LP/P), *TYR* (LP/P)	Yes
**31**	White/NH	No	Nystagmus, fundus hypopigmentation, foveal hypoplasia	Yes	Albinism Sequence Analysis	2016	*OA1/GPR143* (VUS->VUS (reclassification)), *OCA2* (VUS->VUS (reclassification))	No
**32**	White/NH	Yes	Fundus hypopigmentation, foveal hypoplasia, iris TID	Unknown	Hermansky Pudlak and Oculocutaneous Albinism Panel	2013	*OCA2* (LP/P), *OCA2* (LP/P)	Yes
**33**	White/Hispanic	Yes	Nystagmus, fundus hypopigmentation, foveal hypoplasia	Yes	Oculocutaneous Albinism Panel	2020	*OCA2* (P), *OCA2* (P), *GPR143* (VUS)	Yes
**34**	White/NH	No	Nystagmus, fundus hypopigmentation, foveal hypoplasia	Yes	Oculocutaneous Albinism Sequencing Panel	2017	*OCA2* (LP), *TYR* (VUS->haplotype	No

							(reclassification)), *TYR* (VUS)	
**35**	White/NH	Yes	Nystagmus, fundus hypopigmentation, foveal hypoplasia	No	ID my IRD panel	2021	*TYR* (P), *TYR* (LP)	Yes
**36**	White/NH	Yes	Nystagmus, foveal hypoplasia	No	Oculocutaneous Albinism Panel	2021	*TYR* (P), *TYR* (P)	Yes
**37**	Black/NH	Yes	Nystagmus, foveal hypoplasia	Yes	Exome Next Select for OCA Genes	2019	*HPS5* (P), *OCA2* (LP), *OCA2* (VUS)	Yes
**38**	White/NH	No	Foveal hypoplasia, iris TID	No	Invitae Inherited Retinal Disorders Panel	2021	*TYR* (P), *MPDZ* (P), *CDH23* (VUS), *CTNNA1* (VUS), *HMX1* (VUS), *LRP2* (VUS), *ZNF423* (VUS)	No
**39**	Unknown	No	Nystagmus, fundus hypopigmentation, foveal hypoplasia	No	Invitae Inherited Retinal Disorders Panel + WES/Mitochondrial	2021	*OCA2* (P), *ARHGEF18* (VUS), *IDH3A* (VUS), *KIF7* (VUS), *MTTP* (VUS), *SLC7A14* (VUS)	No
**40**	White/NH	No	Nystagmus, foveal hypoplasia	No	Oculocutaneous Albinism Sequencing Panel	2017	*TYR* (VUS), *TYR* (VUS)	No
**41**	Other/Hispanic	Yes	Fundus hypopigmentation, iris TID	Unknown	Albinism Panel by Massively Parallel Sequencing	2014	*OCA2* (LP/P), *HPS6* (VUS->VUS (reclassification))	No
**42**	Unknown	Yes	Nystagmus	Yes	Oculocutaneous Albinism Panel	2020	*TYRP1* (P)	Yes
**43**	Other/Hispanic	Yes	Nystagmus, foveal hypoplasia	No	Albinism Panel (Dermatology) Plus	2018	*TYR* (P), *TYR* (VUS), *HPS4* (VUS), *HPS6* (VUS)	No
**44**	White/NH	Yes	Nystagmus, fundus hypopigmentation, foveal hypoplasia, iris TID	No	Oculocutaneous Albinism Panel	2019	*TYR* (P), *TYR* (VUS), *TYR* (VUS)	Yes
**45**	Black/NH	Yes	Nystagmus, fundus hypopigmentation, foveal hypoplasia	Unknown	Hermansky Pudlak and OCA 15 Gene Panel	2016	*OCA2* (LP/P), *OCA2* (LP/P)	Yes
**46**	Other/Hispanic	Yes	Nystagmus, fundus hypopigmentation, foveal hypoplasia, iris TID	No	HPS1 and HPS3 Genes/ Select Analysis of Common Mutations / Hermansky-Pudlak Syndrome	2008	*HPS5* (LP/P), *HPS5* (LP/P)	Yes
**47**	White/NH	No	Nystagmus, fundus hypopigmentation, foveal hypoplasia	No	Pigmentation Panel through Casey Eye Institute	2014	*TYR* (LP/P), *TYR* (LP/P), *TYR* (VUS), *HPS5* (LP/P)	Yes
**48**	Other/Hispanic	Yes	Nystagmus, foveal hypoplasia, iris TID	Yes	Oculocutaneous Albinism Panel	2019	*TYR* (P), *OCA2* (VUS)	Yes
**49**	Black/NH	Yes	Nystagmus, fundus hypopigmentation, foveal hypoplasia	No	ExomeNext-Select: Analysis of Medical Provider Selected Genes	2020	*OCA2* (P), *OCA2* (LP)	Yes
**50**	Other/Hispanic	No	Nystagmus, fundus hypopigmentation	No	Ocular Albinism, X-linked GPR143 Comprehensive - Sequence & Deletion/Duplication Analysis	2015	*OA1/GPR143* (P)	Yes
**51**	Black/NH	Yes	Fundus hypopigmentation	No	Hermansky Pudlak Syndrome and Oculocutaneous Albinism, CEI	2015	*OCA2* (LP/P), *OCA2* (LP/P)	Yes

					Molecular Diagnostics, RT / NGS Pigmentation SmartPanel			
**52**	White/NH	No	Nystagmus, fundus hypopigmentation, foveal hypoplasia	No	Blueprint Genetics FLEX Albinism Panel (Ophthalmology) Plus	2022	No mutation	No
**53**	White/NH	Yes	Nystagmus, fundus hypopigmentation, foveal hypoplasia	Yes	Invitae Inherited Retinal Disorders Panel	2022	*CACNA2D4* (VUS), *COL18A1* (VUS), *FSCN2* (VUS), *PCDH15* (VUS), *RIMS1* (VUS)	No

“NH” = Not Hispanic. “N/A” = Not Available. “TID” = Transillumination Defect. “P” = Pathogenic. “LP” = Likely Pathogenic. “VUS” = Variants of Uncertain Significance.
“Yes” for Cutaneous findings if characteristic skin findings of albinism were seen
“TID” = transillumination defects
“Yes” for Family History includes patients with family history of cutaneous or ocular manifestations of albinism.
“Yes” for positive diagnostic yield indicates that the patient had genetic testing results that are believed to explain the patient’s ocular or oculocutaneous albinism based on the variant classification and inheritance pattern.
Patient 16 and 28 had reclassifications of VUS’ to P variants that changed the diagnostic yield, Other VUS that underwent reclassification that remained VUS are also listed with “VUS->VUS (reclassification)”
